# GALNT6 expression enhances aggressive phenotypes of ovarian cancer cells by regulating EGFR activity

**DOI:** 10.18632/oncotarget.16585

**Published:** 2017-03-28

**Authors:** Tzu-Chi Lin, Syue-Ting Chen, Min-Chuan Huang, John Huang, Chia-Lang Hsu, Hsueh-Fen Juan, Ho-Hsiung Lin, Chi-Hau Chen

**Affiliations:** ^1^ Department of Obstetrics and Gynecology, National Taiwan University Hospital, Taipei, Taiwan; ^2^ Graduate Institute of Anatomy and Cell Biology, National Taiwan University College of Medicine, Taipei, Taiwan; ^3^ Department of Surgery, National Taiwan University Hospital, Taipei, Taiwan; ^4^ Department of Life Science, Institute of Molecular and Cellular Biology and Graduate Institute of Biomedical Electronics and Bioinformatics, National Taiwan University, Taipei, Taiwan

**Keywords:** ovarian cancer, GALNT6, epidermal growth factor receptor, O-glycosylation, invasion

## Abstract

Ovarian cancer is the most lethal of the gynecologic malignancies. N-acetylgalactosaminyltransferase 6 (GALNT6), an enzyme that mediates the initial step of mucin type-O glycosylation, has been reported to regulate mammary carcinogenesis. However, the expression and role of GALNT6 in ovarian cancer are still unclear. Here we showed that high GALNT6 expression correlates with increased recurrence, lymph node metastasis, and chemoresistance in ovarian endometrioid and clear cell carcinomas; and higher GALNT6 levels are significantly associated with poorer patient survivals. GALNT6 knockdown with two independent siRNAs significantly suppressed viability, migration, and invasion of ovarian cancer cells. Using phospho-RTK array and Western blot analyses, we identified EGFR as a critical target of GALNT6. GALNT6 knockdown decreased phosphorylation of EGFR, whereas GALNT6 overexpression increased the phosphorylation. Lectin pull-down assays with *Vicia villosa* agglutinin (VVA) indicated that GALNT6 was able to modify O-glycans on EGFR. Moreover, the GALNT6-enhanced invasive behavior was significantly reversed by erlotinib, an EGFR inhibitor. Our results suggest that GALNT6 expression is associated with poor prognosis of ovarian cancer and enhances the aggressive behavior of ovarian cancer cells by regulating EGFR activity.

## INTRODUCTION

Epithelial ovarian cancer (EOC) is the leading cause of mortality from cancers of female reproductive tract, because most patients are diagnosed at clinically advanced stages; the 5-year survival rate is less than 45% [1]. EOC is a heterogeneous disease with four major histologic subtypes: serous, endometrioid, clear cell, and mucinous carcinoma [2]. Although each of these subtypes has distinct molecular alterations and differential chemosensitivity, endometrioid and clear cell carcinomas are closely linked to an entity of endometriosis-associated ovarian carcinomas that is etiologically distinguish from other subtypes in several aspects [2]. In Western populations, endometrioid and clear cell carcinomas account for about 15-20% of all EOCs, whereas in East Asia, its prevalence rises to 30-40% [2–5]. The majority of endometrioid carcinomas are low-grade with good prognosis [6]. When confined to the ovary the endometrioid carcinomas have an excellent prognosis, but advanced stage tumors have a poor outcome. Similar to endometrioid carcinomas, clear cell carcinoma commonly presents at an early stage. However, clear cell carcinoma is known to be less sensitive to platinum-based front-line chemotherapy and to be associated with a worse prognosis than serous type EOC [7, 8]. To improve survival of patients with unfavorable ovarian endometrioid or clear cell carcinomas, there is a need to improve our understanding of their pathobiology in order to optimize currently available treatments and develop tailored therapies.

Glycosylation is an enzymatic process that links carbohydrates to molecules, like proteins, lipids, or other sugars, and aberrant glycosylation affects many cellular properties, including cell proliferation, differentiation, transformation, migration, invasion, apoptosis, and immune responses [9–11]. *N*-glycosylation and *O*-glycosylation are the two major types of protein glycosylation in mammalian cells. The most common *O*-glycosylation is the mucin type, initiated by the transfer of N-acetylgalactosamine to the hydroxyl group of serine or threonine residue forming the Tn antigen [12]. This reaction is mediated by a large family of N-acetylgalactosaminyltransferases (GALNTs), consisting of at least 20 members in humans, namely GALNT1 to 20 [12]. Studies have shown that mislocalization and dysregulation of GALNTs expression result in aberrant glycosylation in cancer cells denoting the critical roles of GALNTs in regulating cancer behaviors. For example, dysregulation of GALNT1 contributes to the malignant progression of hepatocellular carcinoma by modifying glycosylation of the epidermal growth factor receptor (EGFR), a member of receptor tyrosine kinase (RTK) family [13]. GALNT2 contributes to malignant behaviors of oral squamous cell carcinoma [14]. GALNT6 modifies Mucin (MUC) 1 glycosylation and regulates proliferation of breast cancer cells [15] and is essential for *O*-glycosylation and stabilization of MUC4 of pancreatic cells [16].

Yet very few studies have explored the role of GALNTs in ovarian cancers. One study showed that GALNT3 is up-regulated in high-grade serous EOC and is correlated with shorter progression-free survival in advanced stage ovarian cancer [17]. Another study suggested that GALNT14 could modulate MUC13 *O*-glycosylation and promote migration of ovarian cancer cells [18]. In the present study, we first reported that higher GALNT6 level is associated with poorer prognosis in patients with ovarian endometrioid or clear cell carcinoma. Moreover, GALNT6 modifies EGFR *O*-glycosylation and plays critical roles in malignant phenotypes of ovarian cancer cells.

## RESULTS

### Increased GALNT6 is associated with decreased survival of endometrioid and clear cell subtypes of ovarian cancer

**Figure 1:**
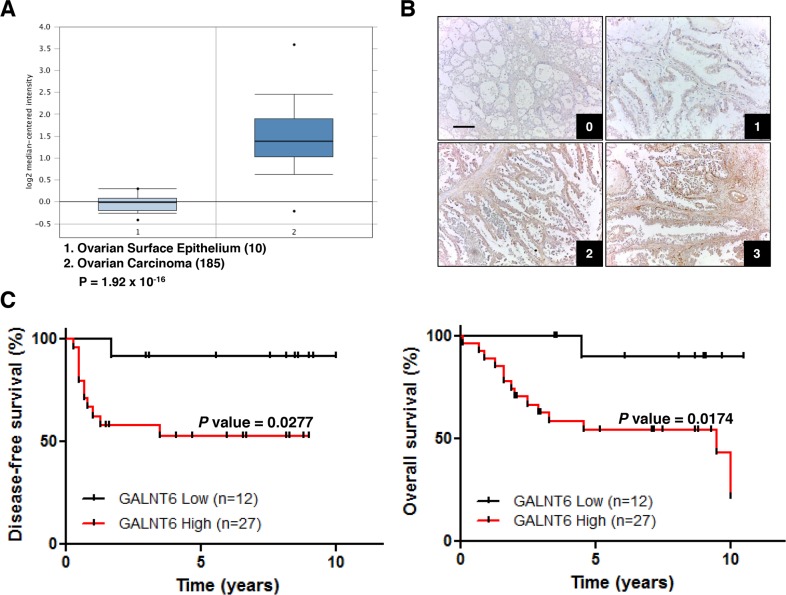
Relationships between GALNT6 expression and clinical features in ovarian carcinomas **(A)** Oncomine database (Bonome Ovarian) shows that *GALNT6* is up-regulated in ovarian carcinoma (n=185) compared with normal ovarian epithelium (n=10). Fold change 2.851, p < 0.001. **(B)** Representative immunohistochemical staining of GALNT6 in clear cell ovarian cancer (0, +1, +2, +3). Scale bar=50 μm. Negative control in clear cell ovarian cancer does not show any specific signals (data not shown). **(C)** Correlation between GALNT6 expression and disease-free survival (left panel) and overall survival (right panel) in patients with endometrioid (n=20) and clear cell (n=19) ovarian carcinoma.

We first examined expression levels of *GALNTs* in ovarian cancers through publically available gene expression datasets. Data retrieved from Oncomine database showed that only *GALNT6, 12, 14* and *15* expression levels were up-regulated in ovarian carcinoma compared with normal ovarian surface epithelium. Among them, *GALNT6* expression levels were markedly up-regulated (Figure 1A). We therefore further analyzed the expression of GALNT6 in ovarian cancerous tissues. The expression levels of GALNT6 were scored as described in the materials and methods. The major clinical characteristics of these patients were examined according to the GALNT6 expression. The survival impact of GALNT6 expression was examined by Kaplan–Meier analysis. Due to the most common histology of EOC is serous, we first examined the GALNT6 expression in 39 patients with ovarian serous carcinoma. However, no significant correlation between the GALNT6 expression and clinicopathological features was found (Supplementary Figure 1A).

Next, tissue blocks from 20 patients with ovarian endometrioid carcinoma and 19 patients with ovarian clear cell carcinoma were selected for immunohistochemical staining. Survival analysis indicated that high GALNT6 expression has a similar trend of poor prognosis in both types of patients (Supplementary Figure 1B and C). According to current histopathologic finding, endometrioid and clear cell carcinomas are closely linked to an entity of endometriosis-associated ovarian carcinomas that is etiologically distinguished from other subtypes in several aspects [2, 19]. Thus, these patients were grouped together for further analysis. In this subgroup, high GALNT6 expression correlated with increased recurrence, lymph node metastasis, and chemoresistance (Table 1). Additionally, Kaplan–Meier analysis indicated that high GALNT6 expression was significantly associated with poor disease-free (Figure 1C, left, *p* = 0.0277) and overall survival (Figure 1C, right, *p* = 0.0174). Collectively, these data revealed that GALNT6 expression is upregulated in ovarian cancer and that increased GALNT6 is associated with poorer prognosis on the endometrioid and clear cell subtypes of ovarian cancer.

**Table 1:** Clinicopathological correlation of GALNT6 expression in endometrioid and clear cell type ovarian cancer (n=39)

Characteristics	GALNT6 expression (No. of patients)			P ^a^
	Low	High	Total	
Age (years)				0.326
≤50	5	7	12	
>50	7	20	27	
Recurrence				0.002
no	11	10	21	
yes	1	17	18	
Chemosensitivity ^b^				0.015
resistance	0	10	10	
sensitive	11	14	25	
Lymph node metastasis ^c^				0.042
no	11	19	30	
yes	0	8	8	
Distant metastasis ^c^				0.354
no	11	25	36	
yes	0	2	2	
Serum CA-125 level^c^				0.003
≤35	6	2	8	
>35	6	24	30	

^a^ Chi-square test

^b^ Four patients did not receive chemotherapy

^c^ One patient did not do preoperative evaluation

### GALNT6 knockdown suppresses malignant phenotypes in ES-2 and OVTW59 cells

**Figure 2:**
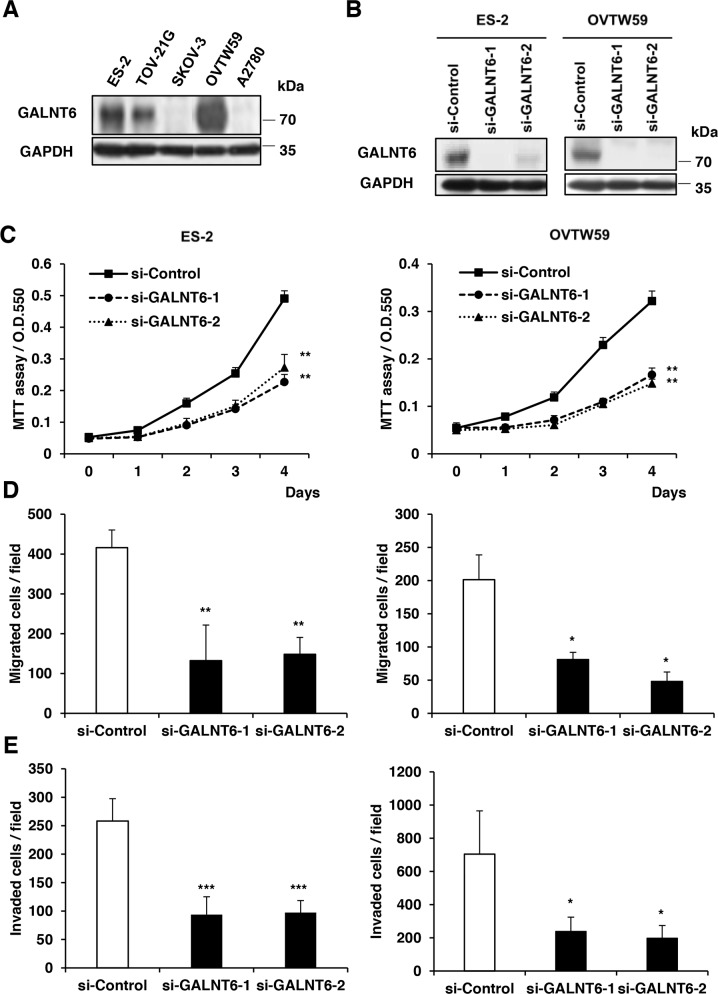
Effects of GALNT6 knockdown on malignant phenotypes in ovarian cancer cells **(A)** GALNT6 expression in ovarian cancer cell lines. GALNT6 was differentially expressed in ES-2, TOV21G, SKOV3, OVTW59, and A2780 by Western blotting. **(B)** Transfection of siRNAs targeting GALNT6 (si-GALNT6-1 and si-GALNT6-2) or control siRNA (si-Control) in ES-2 and OVTW59 cells. The efficiency of GALNT6 knockdown was confirmed by Western blotting. **(C)** Cell viability was analyzed by MTT assay at different time points (Left, ES-2 cells; right, OVTW59 cells). **(D)** Effects of GALNT6 on cell migration in transwell migration assays (Left, ES-2 cells; right, OVTW59 cells). **(E)** Effects of GALNT6 on cell invasion in matrigel invasion assays (Left, ES-2 cells; right, OVTW59 cells). Data are presented as mean ± SD from 3 independent experiments. (*p < 0.05; **p < 0.005; ***p < 0.001).

To study the role of GALNT6 in ovarian endometrioid and clear cell carcinomas, we first analyzed GALNT6 expression in 5 ovarian cancer cell lines (2 endometrioid and 3 clear cells) using Western blotting (Figure 2A). Among these cancer cell lines, ES-2 and OVTW59 cells expressed higher levels of GALNT6, whereas SKOV3 and A2780 cells expressed lower levels of GALNT6. We therefore chose ES-2 cells and OVTW59 cells for siRNA GALNT6 knockdown experiments. GALNT6 knockdown was confirmed via Western blotting (Figure 2B).

To investigate the effects of GALNT6 on malignant phenotypes in ovarian cancer, viability, migration, and invasion were measured in ES-2 and OVTW59 cells with and without GALNT6 knockdown. The MTT assay showed that GALNT6 knockdown decreased the viability of ES-2 and OVTW59 cells (Figure 2C). In addition, migration and invasion were also markedly suppressed in ES-2 and OVTW59 cells with GALNT6 knockdown (Figure 2D and 2E) (*p < 0.05, **p < 0.005, ***p < 0.001).

### GALNT6 regulates EGFR activation via modulating *O*-glycosylation of EGFR

**Figure 3:**
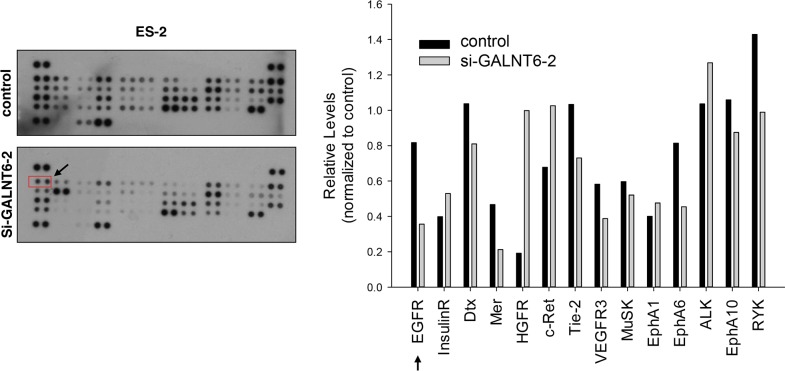
Effect of GALNT6 knockdown on RTK activity Human p-RTK array showing the effect of GALNT6 on RTK phosphorylation. Cell lysates of control and GALNT6 knockdown ES-2 cells were applied to p-RTK arrays. The chemiluminescent film image (left panel) and the quantification of that image (right panel) were shown. The arrows indicate the position of EGFR.

RTK signaling is important for ovarian cancer progression [18], and RTK activity is regulated by *O*-glycosylation [13, 14, 20]. To evaluate the effect of GALNT6 knockdown on RTK activity, a human phospho-RTK array assay including 49 different RTKs was performed (Figure 3, left panel). As shown in right panel of Figure 3, GALNT6 knockdown decreased the phosphorylation of EGFR, Mer, Tie-2, EphA6, VEGFR3, and RYK, in ES-2 cells. Among them, the decrease of phosphorylation was largest in EGFR.

**Figure 4:**
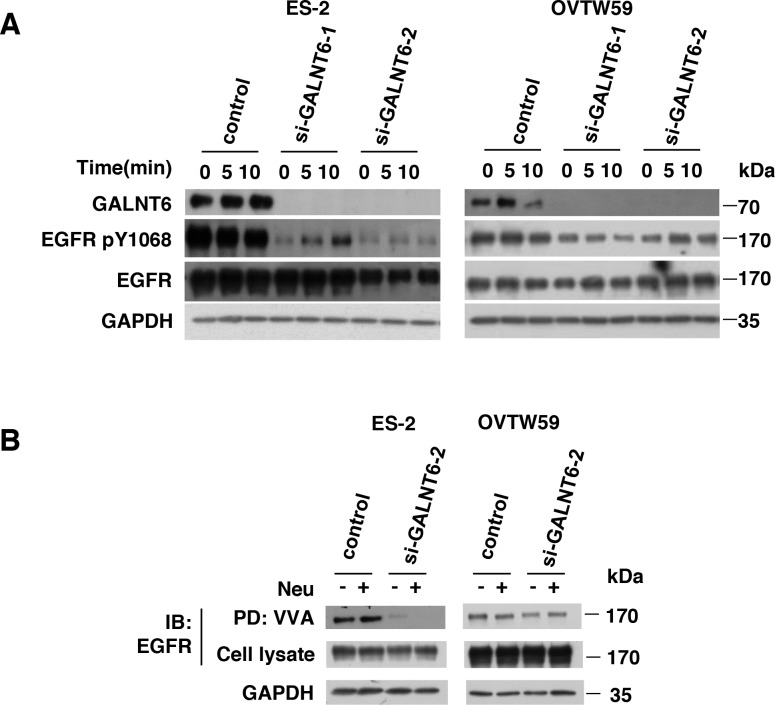
GALNT6 regulate EGFR activation via modulating O-glycosylation of EGFR **(A)** GALNT6 knockdown decreased EGFR phosphorylation in ES-2 and OVTW59 cells. Control and GALNT6-knockdown cells were treated with 10% FBS for 10 minutes, and lysates were analyzed by Western blotting. **(B)** Some ES-2 and OVTW59 lysates were treated with neuraminidase (Neu) to unmask the effects of sialyation, and all were then incubated with VVA-conjugated agarose beads. Proteins pulled down by VVA were analyzed by immunoblotting (IB) with anti-EGFR antibody. Knockdown of GALNT6 decreased VVA binding to EGFR in ES-2 and OVTW59 cells. Total lysate was used as loading control.

We then validated the effect of GALNT6 knockdown on EGFR activation. Total cell lysates were immunoblotted with antibodies for EGFR p-1068 and total EGFR. As shown in Figure 4A, GALNT6 knockdown decreased EGFR phosphorylation in ES-2 and OVTW59 cells. However, flow cytometry showed that GALNT6 knockdown did not alter protein levels of EGFR on cell surfaces (Supplementary Figure 2). To investigate whether GALNT6 can modify O-glycans on EGFR, VVA lectin was used to detect Tn antigen (GalNAc-O-Ser/Thr) expression in the presence or absence of GALNT6 knockdown. To minimize the effects of sialic acids on lectin binding, neuraminidase digestion of the cell lysate was performed. EGFR proteins in control and GALNT6 knockdown ES-2 and OVTW59 cells were immunoprecipitated. As shown in Figure 4B, GALNT6 knockdown reduced VVA binding to EGFR, regardless of neuraminidase treatment. These results indicated that GALNT6 modifies O-glycans on EGFR in ovarian endometrioid and clear cell carcinoma cells.

### Overexpression of GALNT6 enhances malignant behaviors of SKOV3 cells

**Figure 5:**
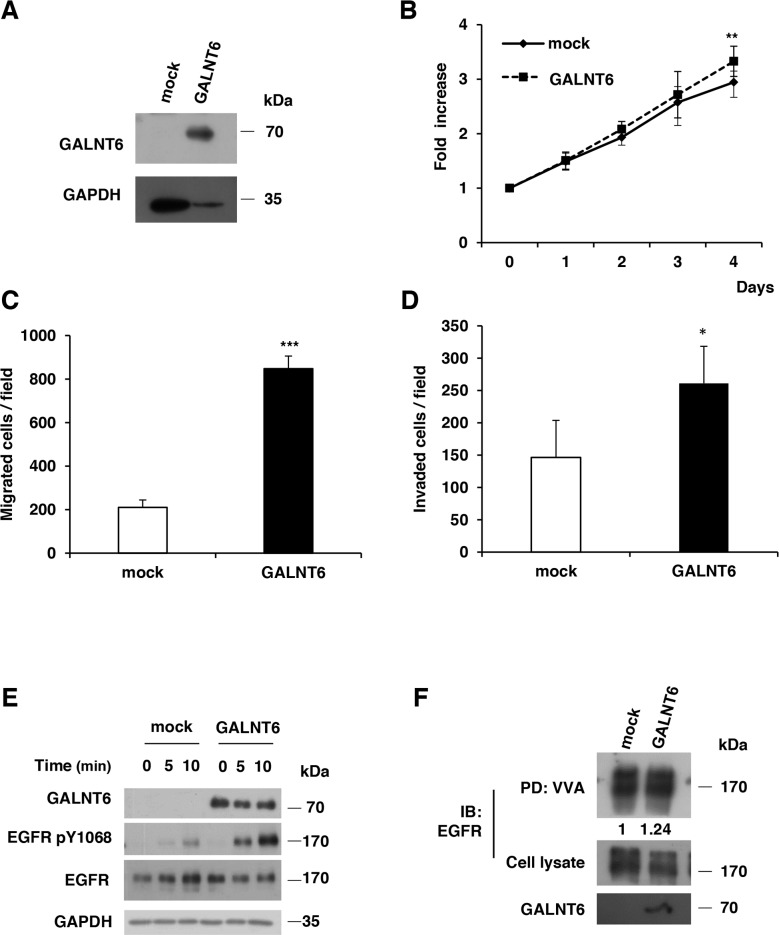
Overexpression of GALNT6 enhances malignant behaviors of SKOV3 cells **(A)** The efficiency of GALNT6 overexpression was confirmed by Western blotting. **(B)** Cell viability was analyzed by MTT assay at different time points. **(C)** Effects of GALNT6 on cell migration in transwell migration assays. **(D)** Effects of GALNT6 on cell invasion in Matrigel invasion assays. **(E)** GALNT6 overexpression enhanced phosphorylation of EGFR. Mock and GALNT6-overexpressed SKOV3 cells were treated with 10% FBS for 10 minutes, and lysates were analyzed by Western blotting. **(F)** Overexpression of GALNT6 increased VVA binding to EGFR in SKOV3 cells. Expressed as integrated intensity normalized to cell lysate (middle panel) quantified by UN-SCAN-IT gel 6.1 software. Data are presented as mean ± SD from 3 independent experiments. (**p < 0.005; ***p < 0.001).

To further confirm the effects of GALNT6 on ovarian cancer cells, GALNT6 was overexpressed in SKOV3 cells (Figure 5A), which expressed low levels of GALNT6. Our results showed that overexpression of GALNT6 enhanced cell viability, migration and invasion (Figure 5B, 5C and 5D). Moreover, overexpression of GALNT6 also increased the phosphorylation of EGFR (Figure 5E) and VVA binding to EGFR (Figure 5F) in SKOV3 cells.

**Figure 6:**
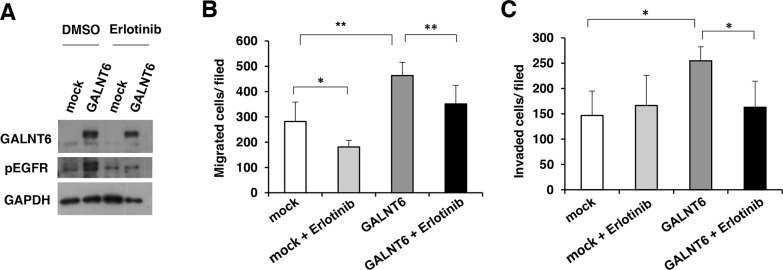
GALNT6-enhanced behaviors are inhibited by EGFR inhibitor **(A)** GALNT6-increased phosphorylation of EGFR was inhibited by erlotinib in SKOV3 cells. **(B)** Transwell migration assays were performed to analyze effects of 5 μM erlotinib on cell migration in mock and GALNT6-overexpressed SKOV3 cells (mock + Erlotinib versus GALNT6 + Erlotinib, p=0.09). **(C)** Matrigel invasion assays were performed to analyze effects of erlotinib on cell invasion in mock and GALNT6-overexpressed SKOV3 cells (mock + Erlotinib versus GALNT6 + Erlotinib, p=0.44). Data are presented as mean ± SD from 3 independent experiments. (*p < 0.05; **p < 0.005).

We also used erlotinib, an EGFR inhibitor, to confirm the significance of EGFR activity in regulating the malignant phenotype of ovarian cancer cells. Our data showed that the GALNT6-increased phosphorylation of EGFR was inhibited by erlotinib in SKOV3 cells (Figure 6A). In addition, the GALNT6-enhanced migration and invasion were significantly blocked by erlotinib (Figure 6B and 6C). Collectively, these results confirm that GALNT6 promotes the aggressive behavior of ovarian endometrioid and clear cell carcinoma cells by regulating EGFR glycosylation and activity.

### GALNT6 knockdown affects gene expression

**Figure 7:**
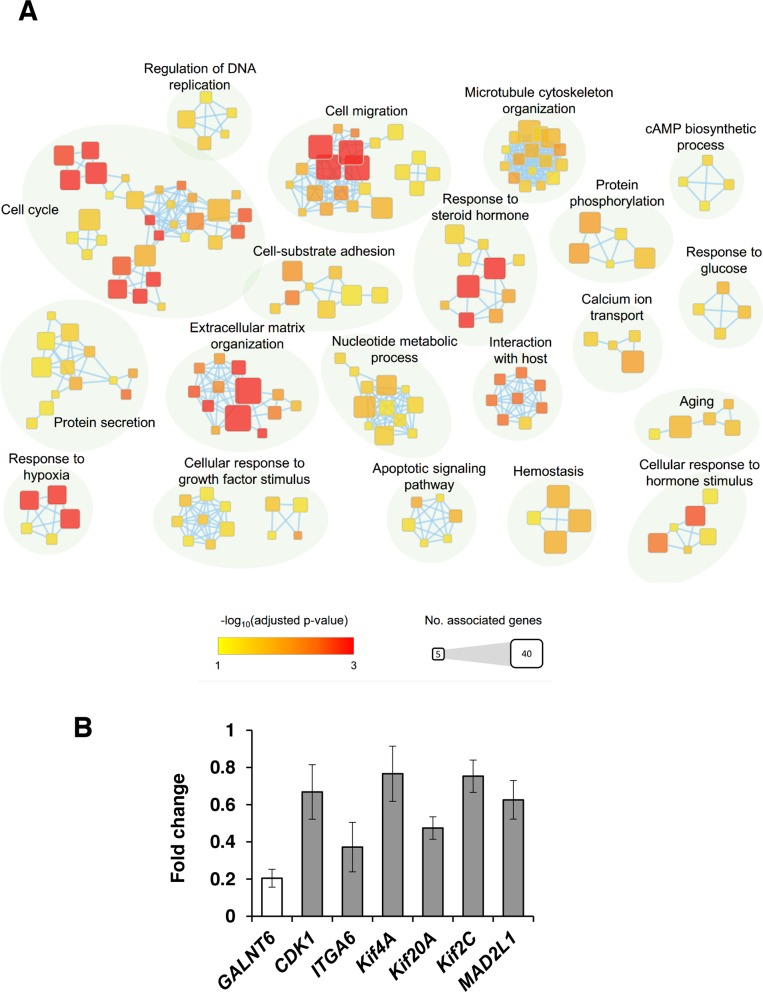
GALNT6 knockdown affects gene expression in ES-2 cells **(A)** Enrichment results of *GALNT6* knockdown in ES-2 cells were mapped as networks. Nodes represent enriched gene sets (p < 0.05), and edges represent gene overlap scores between nodes above the threshold (0.5). Node color encodes the enriched significance and node size is proportional to the number of genes which were associated with a given Gene Ontology term. Groups of functionally related Gene Ontology terms are manually identified and labelled with the appropriate terms. Groups with less than four terms are not shown in this map. **(B)** Quantitative RT-PCR was performed to validate microarray results. Altered expression (fold change) of selected genes upon *GALNT6* knockdown in ES-2 cells compared with control. Values less than 1 represent gene downregulation. Results were analyzed from three independent *GALNT6* knockdown ES-2 cell groups and their corresponding controls and are presented as mean ± SD.

To better understand the molecular mechanisms by which *GALNT6* gene expression affects ovarian cancer progression, we evaluated global gene expression in control and *GALNT6-*knockdown ES-2 cells. All microarray experiments were performed in triplicate where three hybridizations were conducted for each *GALNT6* knockdown cells against the corresponding control. The differential expressions (≥ 1.5-fold change, *p* < 0.05) of functionally related gene groups are provided in Supplementary Table 1. Functional enrichment and network analysis showed that *GALNT6* knockdown in ES-2 cells leads to differential gene expression in various pathways, including cell cycle, cell migration, extracellular matrix organization, response to hypoxia, and response to steroid hormone (Figure 7A). Genes selected for real-time RT-PCR validation are shown in Figure 7B. These results also support that GALNT6 can regulate malignant behaviors of ovarian cancer cells.

## DISCUSSION

Aberrantly expressed O-glycans have been reported in many cancers; however, the roles of GALNT6 in cancers remain largely unclear [15, 16]. In this study, we found that high GALNT6 expression correlates with increased recurrence, lymph node metastasis, and chemoresistance in ovarian endometrioid and clear cell carcinomas; and higher GALNT6 level is significantly associated with poorer patient disease-free and overall survivals. We discovered that GALNT6 knockdown suppressed ovarian cancer cell proliferation, migration and invasion. In contrast, overexpression of GALNT6 enhanced ovarian cancer cell malignant phenotypes. Mechanistic investigation suggests that GALNT6 modifies EGFR *O*-glycosylation, thereby, regulating EGFR phosphorylation, which in turn modulates ovarian cancer cell malignant behaviors. This study is the first to report that GALNT6 plays important roles in malignant behaviors of ovarian cancer.

MUC1 is a transmembrane protein commonly overexpressed in a number of epithelial cancers [21]. Since it has been suggested that GALNT6 could regulate breast cancer cell adhesion and growth through modification of MUC1 glycosylation and stability [15], we therefore tested whether GALNT6 exerted its effects via MUC1 in ovarian cancer cells. We found that, among three ovarian cancer cells in this study, MUC1 protein was only expressed in OVTW59 cells (Supplementary Figure 3A). As expected, VVA pull-down assay showed that GALNT6 could modify *O*-glycans on MUC1 in OVTW59 cells (Supplementary Figure 3B). However, our results indicated that GALNT6 can regulate malignant behaviors not only in OVTW59 but also in ES-2 and SKOV3 cells. Thus, it is very unlikely that GALNT6 enhances the aggressive phenotypes of ovarian cancer cells through the MUC1 pathway.

EGFR plays a critical role in diverse cellular functions such as cellular proliferation, migration, and invasion. Aberrant EGFR expression is detected in up to 60% of EOCs and occurs in all histologic subtypes [22]. Further, aberrant EGFR expression is associated with poor outcome of ovarian cancer patients [23]. In this study, p-RTK array results showed that GALNT6 regulates the phosphorylation of multiple RTKs in ovarian cancer cells, especially EGFR. In addition, knockdown of GALNT6 reduced Tn antigen expression on EGFR and decreased the phosphorylation of EGFR in ovarian cancer cells, suggesting that GALNT6 affects ovarian cancer progression by modifying EGFR activity. Furthermore, an EGFR inhibitor, erlotinib, reversed the effects of GALNT6 overexpression on malignant behavior of ovarian cancer cells, indicating that EGFR is a key mediator of GALNT6 effects on ovarian cancer cells. Our previous studies have shown that the activity of EGFR can be modified by GALNT1 and GALNT2 in hepatocellular carcinoma [13, 20] and by GALNT2 in oral squamous cell carcinoma [14]. This is the first to show the correlation between GALNT6 and EGFR activity in ovarian cancer. It is noteworthy that EGFR may not be the only acceptor substrate of GALNT6. It is likely that, in addition to EGFR, other glycoproteins including surface and secreted molecules may be modified by GALNT6 to cooperatively mediate phenotypic changes in EOC. Glycoproteomic technology will be helpful to further understand the precise mechanisms.

Recent data regarding the genetics and histopathology of EOC has improved our understanding of ovarian carcinogenesis. The so-called EOCs, contrary to what their name suggests, barely originate from the surface epithelium of ovaries [2]. Among the major subtypes of EOC, both endometrioid and clear cell carcinomas are strongly linked to endometriosis [2]. Previous studies have shown that ovarian endometrioid and clear cell carcinomas express the similar genetic mutations, such ARID1A and PI3K [19]. In our study, GALNT6 associated with malignant characteristics is noted in the endometrioid and clear cell subtypes but not in the serous carcinoma. However, Wang et al. reported GALNT3 is up-regulated in high-grade serous EOC and is correlated with shorter progression-free survival in advanced stage ovarian cancer [17]. GALNTs have different, but overlapping, substrate specificities and patterns of expression and are consistently shown to play distinct roles in carcinogenesis and tumor metastasis [24]. GALNT14 modulates death-receptor *O*-glycosylation in pancreatic carcinoma, non-small-cell lung carcinoma, and melanoma cells [25]. GALNT2, sharing a high amino acid sequence homology with GALNT14, regulates the malignant character of hepatocellular carcinoma by modulating the structure of short *O*-glycans on EGFR [20]. Both GALNT1 and GALNT2 could modify *O*-glycosylation of EGFR but exert opposite effects on the activity and stabilization of EGFR [13, 20]. GALNT6 disrupts mammary acinar morphogenesis through *O*-glycosylation of fibronectin [26]. In contrast, *GALNT6* expression in pancreatic cancer is associated with better overall survival [27]. The above data suggest that the different isoforms of the GALNTs family could have distinct roles in tumorigenesis and that even the same GALNT member might have different effects depending on the cellular context. Therefore, assessing GALNTs expression in EOC tumors could provide additional prognostic information. Besides, although it is current practice to treat all subtypes of EOC with the same platinum-based chemotherapy, some subtypes do not respond well to this approach and subtype-specific trials of chemotherapy have been recommended for clear cell carcinoma in particular. Thus, a personalized therapy could be assigned with the use of a GALNT6 inhibitor as a complement to the conventional treatment. This approach may increase a patient's chance to prolong the survival.

Data retrieved from Oncomine database shows that *GALNT6* is overexpressed in EOC samples compared with normal tissues. Thus, although our study found no correlation between GALNT6 expression levels in 39 serous ovarian cancer patients with their corresponding clinicopathological characteristics, perhaps a study on a larger sample size could strengthen the role of GALNT6 as a diagnostic factor of serous type ovarian cancer. However, when we examined a group of patients with ovarian endometrioid and clear cell carcinomas, the high expression of GALNT6 significantly associated with poor outcome. Thus, it is tempting to speculate that GALNT6 confers a more aggressive phenotype to endometrioid and clear cell ovarian cancer cells. To better elucidate the molecular mechanisms and biological pathways implicated in GALNT6-mediated effects in ovarian cancer cells, we used a cDNA microarray technology to analyze changes in gene expression and identify the molecular targets upon *GALNT6* suppression. The gene expression data and consecutive functional enrichment and network analysis further support the data obtained by the clinicopathological characteristics, showing that high GALNT6 expression correlates with increased recurrence, lymph node metastasis, and chemoresistance. Indeed, *GALNT6* knockdown resulted in reduced expression of genes associated with cell cycle, cell migration, extracellular matrix organization, and response to hypoxia (Figure 7A and Supplementary Table 1). However, further studies are still needed to verify the role of GALNT6 in the chemoresistance of endometrioid and clear cell ovarian carcinomas.

In conclusion, high GALNT6 expression is associated with poor prognosis in ovarian endometrioid and clear cell carcinomas. Knockdown of GALNT6 suppresses malignant phenotypes in ovarian cancer cells. Furthermore, GALNT6 knockdown decreases EGFR activation and reduces Tn antigen expression on EGFR. These findings suggest that GALNT6 enhances ovarian cancer progression by modifying EGFR *O*-glycosylation and phosphorylation.

## MATERIALS AND METHODS

### Patient samples and cell lines

Following written consent obtained from patients before sample collection and approval by our Institutional Review Board, paraffin embedded archived tissue blocks from 78 patients (39 serous, 20 endometrioid and 19 clear cell carcinomas) treated at the National Taiwan University Hospital between 2004 and 2007 were selected for the study.

Five ovarian cancer cell lines, ES-2 (American Type Culture Collection; ATCC), SKOV3 (ATCC), TOV21G (ATCC), A2780 (ATCC) and OVTW59 (a kind gift from Dr. P. L. Tong, Department of Obstetrics and Gynecology, National Taiwan University), were used in this study. ES-2, SKOV3 and TOV21G are clear cell cancer cell lines, whereas A2780 and OVTW59 are endometrioid cancer cell lines [28, 29]. All were cultured at 37°C in 5% CO2 in DMEM supplemented with 10% fetal bovine serum (FBS; PAA Laboratories).

### Tissue immunohistochemistry

For immunohistochemical staining, Paraffin-embedded human ovarian sections were probed with GALNT6 polyclonal (1:200, Sigma) antibody diluted with 5% BSA/PBS for 16 hours at 4°C, and stained using the Super Sensitive Link- Label immunohistochemistry Detection System (BioGenex). All sections were counterstained with hematoxylin. Negative controls were done by replacing primary antibody with control IgG. The intensity of GALNT6 staining was quantified by a microscope-based image analysis program (Image Pro Plus; Media Cybemetics, Silver Spring, MD). At least three random fields in each section were examined and analyzed at 100x magnification. A semi-quantitative immunoreactivity scoring (IRS) system was applied to assess the immunostaining. Immunostaining intensity (I) was graded as 0 (no staining), 1 (weak staining), 2 (moderate staining), or 3 (strong staining). The percentage of immuno-reactive cells (P) was graded as 0 (none), 1 (<10%), 2 (10–50%), 3 (51–80%), or 4 (>80%). Multiplication of I and P resulted in an IRS ranging from 0 to 12 for each tumor. We used a grouping algorithm (raw scores, low [IRS 0–6] *vs*. high [IRS 7–12]) to test the correlation between GALNT6 expression and clinicopathologic features in ovarian carcinoma patients.

### siRNA knockdown of GALNT6 expression

In transient knockdown experiments, two siRNA oligonucleotides against GALNT6 and a non-targeting siRNA control were synthesized by Invitrogen. ES-2 and OVTW59 cells were transfected with siRNA using Lipofectamine RNAiMAX (Invitrogen) with a final concentration of 1 nM.

### Overexpression of GALNT6 in ovarian cancer cells

The RT-PCR products of full-length human *GALNT6* (Accession No. NM_007210) were cloned into pcDNA3.1/myc-His (Invitrogen Life Technologies) to generate the *GALNT6*/myc-His fusion gene. The insert was confirmed by DNA sequencing. Overexpression of the *GALNT6* gene was achieved by transfecting SKOV3 cells with pcDNA3.1/*GALNT6*/mycHis plasmids using Lipofectamine 3000 (Invitrogen, Life Technologies) according to the manufacturer's protocol. The transfected cells were selected with 500 μg/mL of G418 for 14 days and then pooled for further studies.

### Western blot analysis

Total cell lysates from cultured cells were used. Equal amounts (30 μg) of extracted protein were resolved on SDS-PAGE by electrophoresis, transferred and blocked in TBST (20 mM Tris–HCl, 137 mM NaCl, and 0.1% Tween 20, pH 7.5). The polyvinylidine difluoride membrane was incubated with primary antibody overnight at 4°C, and then with horseradish peroxidase conjugated secondary antibody for 1 hour. The primary antibodies used were GALNT6 (Sigma), GAPDH (Meridian Life Science), EGFR and EGFR pY1068, (Cell Signaling Technology, Inc.). Specific bands were detected by enhanced chemiluminescence detection system (Amersham, Uppsala, Sweden). Protein signals were quantified by optical density ratios using GAPDH expression as a control.

### Lectin pull down assay

*Vicia villosa* Lectin (VVA) agarose beads (Vector Laboratories) were used to detect the Tn antigen on glycoproteins. Cell lysates (0.5 mg) were incubated with or without neuraminidase, an enzyme that removes sialic acids, at 37°C for 1 hour, and then applied to VVA-conjugated agarose beads at 4°C for 16 hours. Precipitated proteins were then used for Western blotting.

### Cell viability

The cell viability was assessed by measuring the ability of cells to reduce 3-(4,5-dimethylthiazol-2-yl)- 2,5-diphenyltetrazolium bromide (MTT) to the dark blue formazan product. According to the manufacturer's instructions (Cayman Chemical, Ann Arbor, MI), ovarian cancer cells were seeded at a density of 1 × 10^3^ cells per well and incubated with MTT for 4 hours at 37°C. Absorbance was read at 550 nm.

### Transwell migration assay

Cell migration was evaluated in 24-well transwell culture chambers. The siGALNT6-transfected cells (1 × 10^4^) were re-suspended in serum-free DMEM and added to the upper well of each migration chamber with an 8-μm pore size membrane (Corning). Cell migration was induced by 10% FBS in the lower chamber. After 24 hours, cells that migrated to the lower surface of the filter were stained with 0.5 % (wt/vol) crystal violet (Sigma) and counted. In some experiments, cells were pretreated with 5 μM erlotinib (Santa Cruz Biotechnology, Santa Cruz, CA) or 0.02% dimethyl sulfoxide (DMSO) for 48 hours to inhibit EGFR activity.

### Matrigel invasion assay

Cell invasion assays were done in BioCoat Matrigel invasion chambers (Becton Dickinson) according to the manufacture's protocol. Briefly, DMEM with 10% FBS as a chemoattractant was loaded in the lower part of the chamber, and 1 × 10^4^ transfected cells in 200 μL serum-free DMEM were seeded onto the upper part. Cells were allowed to invade the matrigel for 24 hours. Invading cells were fixed and stained with 0.5% (wt/vol) crystal violet. Cell numbers were counted for each well, and values are presented as mean ± SD.

### Phospho-receptor tyrosine kinase array

A human phospho-receptor tyrosine kinase (p-RTK) array kit including 49 RTKs was purchased from R&D systems. si-Control and si-GALNT6-transfected ES-2 cancer cells were cultured until confluence on 10 cm^2^ culture plates. Cells were lysed and 250 μg of protein were used for Western blotting according to the manufacturer's protocol. Activated receptors were matched according to the phospho-RTK array coordinates.

### Gene expression profiling and quantitative RT-PCR validation

Total RNA from control and GALNT6 siRNA knockdown ES-2 cells was extracted in triplicates using GeneJET RNA Purification kit (Thermo Scientific) following the manufacturer's protocol and quantified by NanoDrop spectrophotometer (Bio- Rad). The RNA quality was monitored with Agilent 2100 Bioanalyzer (Agilent Technologies, Santa Clara, CA). cDNA prepared from 10 μg of total RNA was labeled with aa-dUTP using Invitrogen SuperScriptTM Plus Indirect cDNA Labeling System according to the manufacturer's protocol, followed by aa-cDNA column purification (QIAGEN, Valencia, CA). Alexa/CyDye was incorporated to aa-cDNA followed by column purification with Alexa/CyDye-cDNA cRNA purification (Qiagen). DNA yields were confirmed by 1% DNA agarose gel and visualized with Fuji image reader at 600V PMT. Agilent Gene Expression Hybridization Kit was used for hybridization according to the manufacturer's instruction. Briefly, 16 ul of dye labeled cDNA were hybridized to Agilent SurePrint G3 Human Gene Expression 8×60K v3 Microarray. The microarrays were scanned on the Agilent DNA Microarray Scanner (US9230696) using one color scan setting for 8×60k array slides. The scanned images were analyzed with Feature Extraction Software 10.5.1.1 (Agilent). Features flagged in Feature Extraction as Feature Non-uniform outliers were excluded. Quantitative RT-PCR of selected genes expression was performed for validation as described above. The primers were designed with Primer3 (v.0.4.0) algorithm with the sequences freely available from the Entrez Nucleotide database. All microarray experiments were performed in triplicates where three hybridizations were conducted for each *GALNT6* knockdown cells against the corresponding control. The microarray data were deposited in the GEO database, accession number GSE89639.

### Function enrichment analysis

The function enrichment analysis was applied to the differentially expressed genes. The functional gene sets were obtained from Gene Ontology (GO). The Fisher's exact test was used to assess if a GO term was enriched in differentially expressed genes. P-values were corrected by the Benjamini-Hochberg method. The enriched GO terms (corrected *p*-value < 0.05) were graphically organized into a network, where a node denotes each GO term and an edge represents gene overlap between GO terms. The gene set overlap was scored by the arithmetic average of Jaccard coefficient $\text{JC}=\frac{\left|A\cap B\;\right|}{\left|A\cup B\;\right|}$ and Simpson coefficient $\text{SC}=\frac{\left|A\cap B\;\right|}{\min(\left|A\;\right|,\left|B\;\right|)}$ in which *A* and *B* are two gene sets. An edge with overlap score > 0.5 was presented in the networks. The networks were visualized by Cytoscape [30].

### Statistical analyses

Statistical analyses were performed using the SPSS 22.0 (SPSS Inc, Chicago, IL) statistical software package. The relationships between GALNT6 expression and clinicopathologic characteristics were tested using Chi-square test. Survival curves were plotted by Kaplan-Meier method and compared by log-rank test. Student's t-test was used to analyze in vitro experiments in cell lines. Data are presented as means ± SD. *p* < 0.05 or less was considered to be statistically significant, and all experiments were performed in triplicate to verify reproducibility.

## SUUPLEMENTARY FIGURES AND TABLE




