# Supplementary material for: GALNT6 expression enhances aggressive phenotypes of ovarian cancer cells by regulating EGFR activity
Source: Oncotarget. 2017 Mar 28;8(26):42588–601. doi: 10.18632/oncotarget.16585 (PMC5522090; doi:10.18632/oncotarget.16585)
Supplement: Supplementary file 1 [file oncotarget-08-42588-s001.pdf]

GALNT6 expression enhances aggressive phenotypes of ovarian cancer cells by regulating EGFR activity

SUUPLEMENTARY FIGURES AND TABLE

(A) Serous type (n=39)

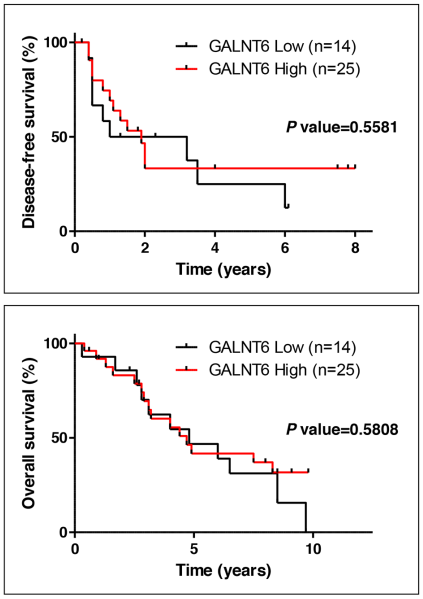

Serous type ovarian cancer				
Characteristics	GALNT6 Staining Intensity (No. of patients)			*P* value
	Low	High	Total	
**Age (years)**				0.303
≤50	7	9	16	
>50	7	16	23	
**Recurrence**				0.507
no	2	5	7	
yes	12	20	32	
**Chemosensitivity**				0.393
resistance	7	10	17	
sensitive	7	15	22	
**Lymph node metastasis** [a]				0.501
no	5	11	16	
yes	2	7	9	
**Distant metastasis**				0.297
no	10	21	31	
yes	4	4	8	
**Debulking**				0.416
optimal	6	13	19	
worst	8	12	20	
**Serum CA-125 level** [b]				0.735
≤35	1	2	3	
>35	12	23	35	

[a] Fourteen patients with no preoperative data
[b] One patient with no preoperative data

(B) Clear cell type (n=19)

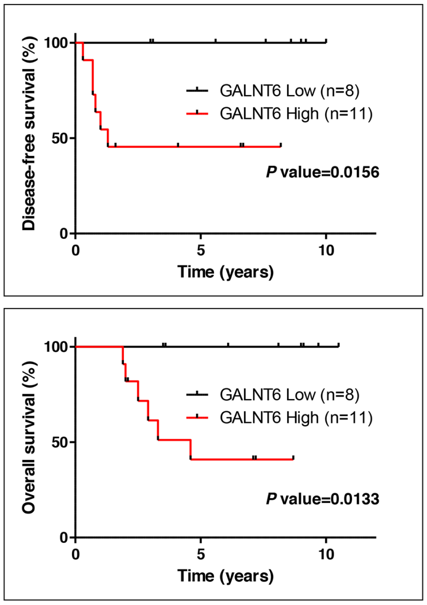

Clear cell type ovarian cancer				
Characteristics	GALNT6 Staining Intensity (No. of patients)			*P* value
	Low	High	Total	
**Age (years)**				0.297
≤50	4	3	7	
>50	4	8	12	
**Recurrence**				0.017
no	8	5	13	
yes	0	6	6	
**Chemosensitivity** [a]				0.054
resistance	0	5	5	
sensitive	7	6	13	
**Lymph node metastasis**				0.322
no	8	9	17	
yes	0	2	2	
**Distant metastasis**				0.579
no	8	10	18	
yes	0	1	1	
**Debulking**				0.17
optimal	8	8	16	
worst	0	3	3	
**Serum CA-125 level**				0.166
≤35	4	2	6	
>35	4	9	13	

[a] One patient did not do chemotherapy

Supplementary Figure 1: Relationships between GALNT6 expression and clinical features in different subtypes of ovarian cancer. **(A)** Correlation between GALNT6 expression and disease-free survival (left upper panel) and overall survival (left lower panel) and association of clinicopathologic variables with GALNT6 expression (right panel) in 39 patients with serous type ovarian carcinoma. **(B)** Correlation between GALNT6 expression and disease-free survival (left upper panel) and overall survival (left lower panel) and association of clinicopathologic variables with GALNT6 expression (right panel) in 19 patients with clear cell type ovarian carcinoma. (*Continued*)

(C) Endometrioid type (n=20)

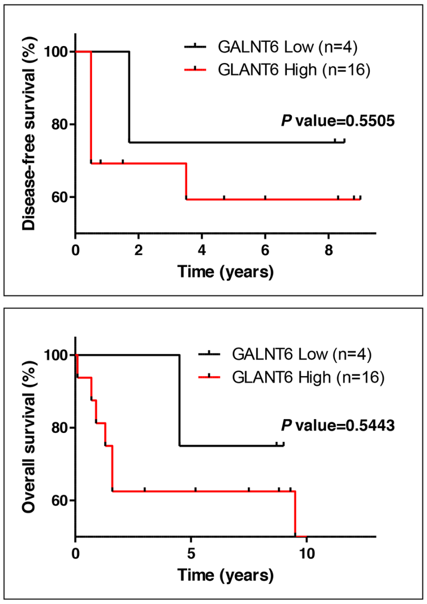

Endometrioid type ovarian cancer

Characteristics	GALNT6 Staining Intensity (No. of patients)			P value
	Low	High	Total	
Age (years)				0.657
≤50	1	5	6	
>50	3	11	14	
Recure				0.153
no	3	5	8	
yes	1	11	12	
Chemosensitivity [a]				0.208
resistance	0	5	5	
sensitive	4	8	12	
Lymph node metastasis [b]				0.295
no	3	10	13	
yes	0	6	6	
Distant metastasis [b]				0.842
no	3	15	18	
yes	0	1	1	
Debulking				0.376
optimal	4	12	16	
worst	0	4	4	
Serum CA-125 level [b]				0.035
≤35	2	0	2	
>35	2	15	17	

[a] Three patients did not do chemotherapy
[b] One patient with no preoperative data

Supplementary Figure 1: (*Continued*) Relationships between GALNT6 expression and clinical features in different subtypes of ovarian cancer. (C) Correlation between GALNT6 expression and disease-free survival (left upper panel) and overall survival (left lower panel) and association of clinicopathologic variables with GALNT6 expression (right panel) in 20 patients with endometrioid type ovarian carcinoma.

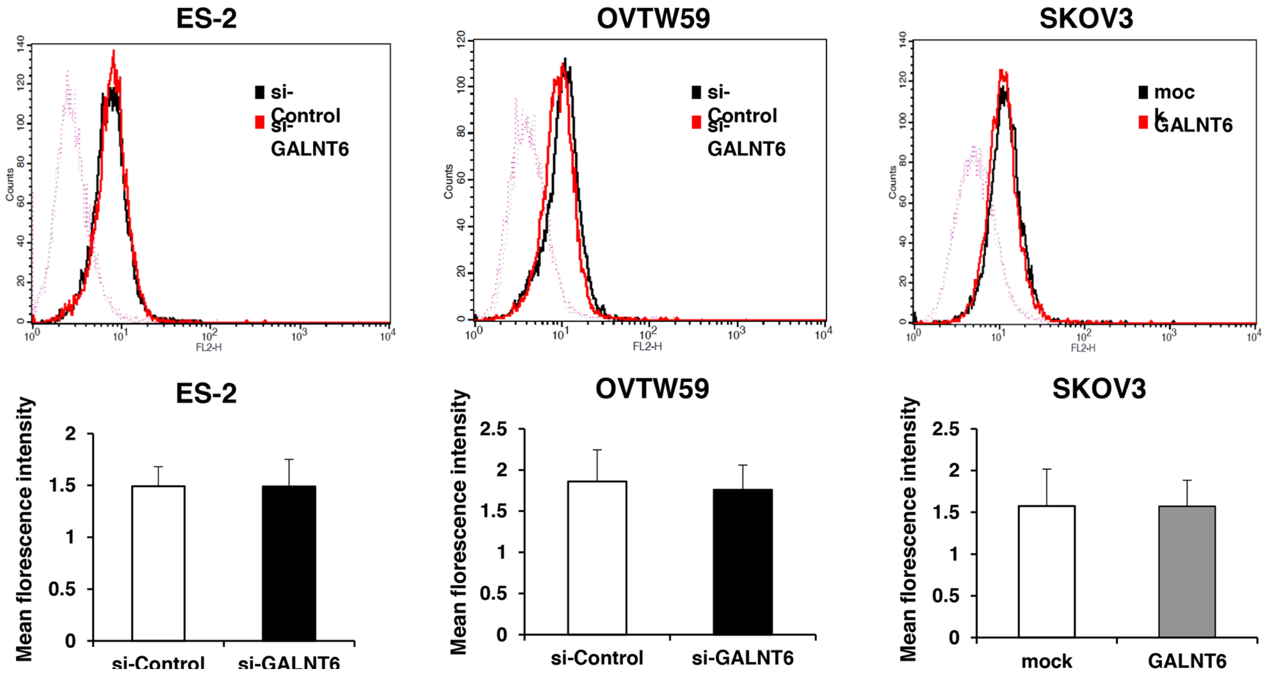

Supplementary Figure 2: Flow cytometry of EGFR on ovarian cancer cell surfaces. ES-2, OVTW59 and SKOV3 cells were detached with 10 mM EDTA and resuspended in PBS with 2% BSA. Cells (1×10^5/100 µl) were incubated with EGFR primary antibodies (sc-120/ Senta Cruz) at 1×100 dilutions on ice for 30 minutes. Cells were washed twice with ice cold 2% BSA/PBS and then incubated with PE-conjugated secondary antibody on ice for 15 min. The fluorescence intensity of 1×10^4 cells for each sample was analyzed. Secondary antibody only was used as control. The mean florescence intensity was averaged from three independent experiments. The representative images are shown (upper). Data are presented as mean ± SD from 3 independent experiments (lower).

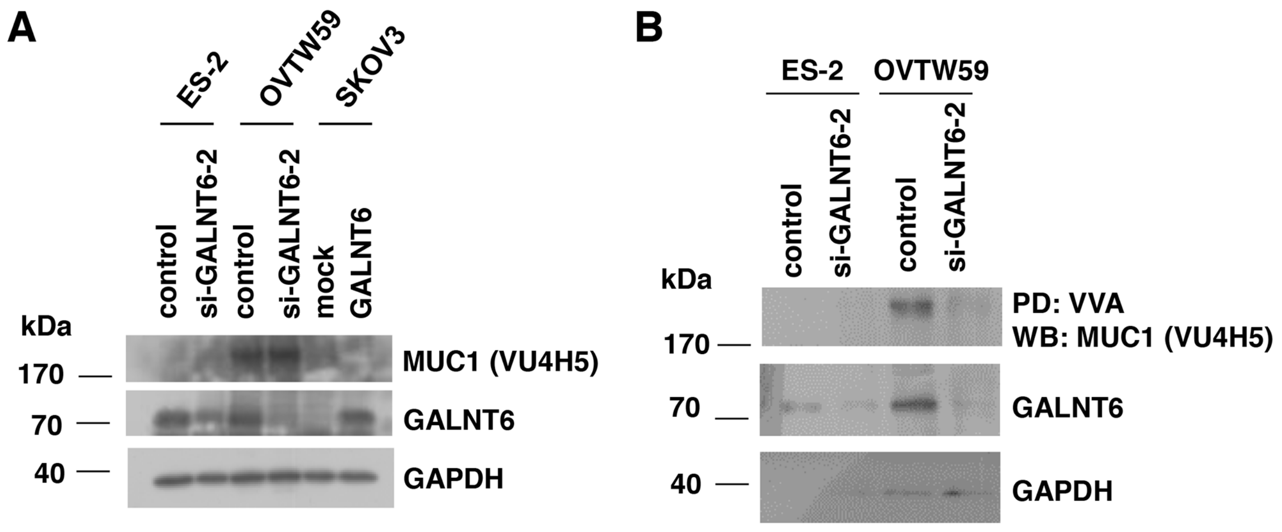

Supplementary Figure 3: MUC1 and its Tn antigen expression in ovarian cancer cells. **(A)** Western blots showing only OVTW59 cells expressed detectable levels of MUC1 as revealed by an anti-MUC1 monoclonal antibody (clone VU4H5). Ovarian cancer cells were transfected with control siRNA or si-GALNT6-2 as well as empty vector (mock) or GALNT6 plasmid. **(B)** *Vicia Villosa* lectin (VVA) pull-down assay showing Tn antigen on MUC1 was decreased by GALNT6 siRNA (si-GALNT6-2) compared with control siRNA in OVTW59 cells. Cell lysates were pulled down (PD) by VVA beads and then Western blotted (WB) with anti-MUC1 monoclonal antibody (VU4H5). GAPDH was used as loading control.

Supplementary Table 1: Selected differentially expressed gene groups in ES-2 cells upon GALNT6 knockdown.

See Supplementary File 1
