# Supplementary material for: GALNT6 expression enhances aggressive phenotypes of ovarian cancer cells by regulating EGFR activity
Source: Oncotarget. 2017 Mar 28;8(26):42588–601. doi: 10.18632/oncotarget.16585 (PMC5522090; doi:10.18632/oncotarget.16585)
Supplement: Supplementary file 2 [file oncotarget-08-42588-s002.docx]

**Supplementary Table 1: Selected differentially expressed gene groups in ES-2 cells upon GALNT6 knockdown.**

|  | **Downregulated genes** |   **Upregulated genes**   |
| --- | --- | --- |
| **Extracellular matrix organization** | AGRN, AGRN, ITGA6, FBN2, ADAM15, DDR1, COL14A1 | COL13A1, MMP14, P4HA1, ITGA5, MMP2, ITGA1, PXDN, FBN1, ICAM1, ITGB3, FOXC2, ITGB5, THBS1, SERPINH1, VCAM1, ERO1B, COL6A3, COL5A2, COL7A1, LAMC1, LAMB3, RECK, ADAMTS4, P3H2, COL1A1, TGFBI, MFAP3, SPARC, PLOD2, DDR1, FN1, COL14A1, COL5A1, ITGAV, CTSB, BMP1  CTSB, BMP1 |
| **Cell migration** |   BCL2, PRKD1, PTPRZ1, ARHGEF39, MYADM, FGF7 | BDKRB1, MMP14, P2RY6, ITGA5, ATOH8, SEMA3C, ITGA2, IL1B, ICAM1, CXCL12, ITGB3, FOXC2, F2RL1, WNT5B, F3, NRP1, CCL20, COL1A1, PTGER4, ARHGEF39, IL23A, PDGFRA, SPARC, PLAU, CD274, CSF1, FN1, IL6, PODXL, ITGAV, F2R |
|    **Cell cycle G1/S phase transition**    | TYMS, CCND1, MCM3, DHFR, CDC45, PCNA, IQGAP3, PRIM1, CDC7, CDK3, DBF4, CDKN3, PPP2R3B | PLK3, LATS1, RPA1, INHBA, CDK3, LATS2, PSMD9 |
| **Cell division**    | CCND1, MAD2L1, SPC25, PIK3CB, PPP2R2D, MAPRE1, TRIOBP, HAUS4, RECQL5, CDK3, ARPP19, CDCA3, HAUS7, NDC80, KIFC1, HELLS, | PPP2R2D, TRIOBP, CDK3, LATS1, LATS2, CCNG2, MAU2 |
|  | CDC7, BIRC5, KATNB1, FAM83D, NEK1, USP44, NEDD9, CENPV, SPAG5, TIMELESS, SKA2, LIG3 |   |
| **Response to hypoxia** | BCL2, LONP1, HYOU1 | CA9, MMP14, CXCL12, ITGA2, STC1, IL1B, ICAM1, CASP1, NR4A2, PML, THBS1, VCAM1, STC2, BIRC2, MMP2, VASN, ITPR1, ECE1, NDRG1, RORA, PLAU, PLOD2, CPEB2, ANGPTL4 |
| **Response to steroid hormone** | CCND1, BCL2, DNMT1, CAD, TYMS, GJB2, BRCA2, CLDN4, CALM3 |   CA9, MMP14, AHR, HEY1, ITGA2, STC1, IL1B, THBS1, F3, NPC1, SMAD6, NR3C1, ERRFI1, GBA, COL1A1, PTGER4, PTGFR, PTGER2, TIMP3, JUNB, SPARC, FN1, FBXO32, STAT3, ,IL6 |
| **Cell junction organization** | BCL2, GJB2, MPP7, FLNA, CNTNAP1, ITGA6, CDH5, MYADM, PKP3   |   SMAD7, LAMC1, LAMB3, FN1, ITGB3, PVR, PTPRK, ITGA5, LIMS1, ITGA2, CDH6, CLDN14 |
